# Medicinal Resins of Socotra: Phytochemical Diversity, Biological Activities, Safety, and Evidence Gaps

**DOI:** 10.3390/molecules31213904

**Published:** 2026-10-29

**Authors:** Abeer Essam Noman, Naif S. Al-Barha, Fuad A. Abdu

**Affiliations:** 1 School of Medical Sciences, Shandong Xiehe University, Jinan 250109, China; 2 Department of Physical Activity, Faculty of Education and Science, Rada’a, Albaydha University, Albaydha 14517, Yemen

**Keywords:** Socotra, *Dracaena cinnabari*, *Boswellia*, *Commiphora*, medicinal resins, oleo-gum resin, phytochemistry, natural products, ethnopharmacology, toxicity

## Abstract

Socotra (Yemen) is home to several resin-producing plant groups. This review evaluates the phytochemistry, biological activities, and safety of *Dracaena cinnabari*, Socotran *Boswellia*, and *Commiphora* resins, identifies gaps in the available evidence, and distinguishes resin-derived findings from bark-derived evidence. *D. cinnabari* resin contains homoisoflavans and chalcones, but quantitative data for major phenolics remain insufficient. Resin preparations showed antifungal and radical-scavenging activity in vitro; a resin extract and two chalcone derivatives reduced hepatitis B virus antigen production in culture without establishing antiviral efficacy. A methanolic resin extract was cytotoxic to six oral squamous cell carcinoma lines (half-maximal inhibitory concentration [IC_50_]: 5.9–19.2 µg/mL). Two rat carcinogenesis protocols reported 14.3% carcinoma incidence at 1000 mg/kg versus 85.7% in carcinogen-only controls. A single study of acute and 28-day oral toxicity provides limited, extract-specific safety data. *Boswellia* and *Commiphora* resin chemistry is documented mainly through volatile profiles. *B. elongata* resin yielded 21 compounds, including boswellic acids, whose presence does not establish pharmacological equivalence to *B. serrata* or *B. sacra*; four triterpenoid acids inhibited yeast α-glucosidase (IC_50_ 9.9–56.8 µM), but antidiabetic efficacy was not tested. Most other *Boswellia* bioactivity data concern bark. (+)-Yangambin accounted for 3.50–9.05% (*w*/*w*) of recovered *C. ornifolia* ethanol extracts. No eligible bioassays of *Commiphora* resins or human studies were identified. The evidence does not support therapeutic claims.

## 1. Introduction

The Socotra Archipelago (Yemen), in the north-western Indian Ocean, was inscribed on the United Nations Educational, Scientific and Cultural Organization (UNESCO) World Heritage List in 2008 primarily for its flora. Of the approximately 825 plant species recorded, 307 (37%) are considered endemic [1]. Several endemic trees yield resinous exudates with documented roles in Socotran ethnomedicine: the red resin of *Dracaena cinnabari* Balf.f. (dragon’s blood) and the oleo-gum resins of *Boswellia* (frankincense) and *Commiphora* species [2,3]. These three groups were selected because their medicinal resin use is documented in Socotran ethnobotanical literature and their available species-specific studies permit a critical comparison of phytochemical evidence, reported biological activities, and research gaps [2,3,4].

The botanical sources, documented traditional uses, and reported major chemical classes are summarised in Table 1. However, the available evidence has not been adequately distinguished according to botanical identity, plant organ, and preparation type, making it difficult to determine which phytochemical, biological, and safety findings are directly supported by studies of Socotran resins. The terminology used in this review distinguishes collected plant material from the preparations subsequently analysed. “Resin” refers to the collected plant exudate. Dragon’s blood from *D. cinnabari* is a red, phenolic-rich resin, whereas the exudates of *Boswellia* and *Commiphora* are generally described as oleo-gum resins, comprising resinous, volatile-oil and gum fractions. “Oleoresin” denotes a resinous material containing volatile oil without necessarily implying a gum fraction and is not used interchangeably with “oleo-gum resin”. “Resin extract” and “bark extract” refer to solvent extracts prepared from the respective materials. “Resin essential oil” and “bark essential oil” refer to volatile oils obtained by distillation from resin and bark, respectively. Headspace volatiles, sampled from the vapour above a material, are distinguished from distilled essential oils. Findings obtained from these different preparations are not considered interchangeable.

The Burseraceae are unusually diverse on the archipelago. The archipelago supports 12 endemic *Boswellia* species and five *Commiphora* species, four of which are endemic to Socotra. By 2017, resin chemistry had been published for only four of the Burseraceae species [4]. This matters for interpretation, because most analytical and pharmacological work on commercial frankincense and myrrh concerns continental taxa. For example, a comparative high-performance liquid chromatography–tandem mass spectrometry (HPLC–MS/MS) study of boswellic and lupeolic acids in 41 oleo-gum resins covered nine *Boswellia* species, none of them Socotran [5]. Findings from such taxa cannot be assumed to hold for Socotran species.

The same caution applies to dragon’s blood. The name is applied to red resins from several genera and species [6], and much of the pharmacological literature concerns *Dracaena cochinchinensis* and other taxa used in Chinese medicine rather than *D. cinnabari* [7]. Resin availability also faces conservation constraints. *Dracaena cinnabari* is listed as Vulnerable on the International Union for Conservation of Nature (IUCN) Red List; however, its latest published global assessment dates to 2004 and is marked as “Needs updating” by the IUCN [8]. A forest inventory of 819 trees in Firmihin found that a tapping technique that removes bark together with the resin had left 84% of trees wounded; the traditional management system was judged unsustainable [9].

**Table 1:** Botanical sources, documented traditional uses and major chemical classes of Socotran medicinal resins. The material analyzed is specified because several studies used bark rather than resin.

Taxon	Status	Material Analyzed	Documented Traditional Use	Major Chemical Classes Reported	References
*Dracaena cinnabari* Balf.f. (Asparagaceae)	Endemic; IUCN Vulnerable	Resin	Topical: dermal, dental and eye conditions, external bleeding. Oral: gastrointestinal complaints, internal and postpartum bleeding	Homoisoflavans, flavans, chalcones, dihydrochalcones, bi- and triflavonoids, metacyclophane, flavylium chromophore, sterols, volatile terpenoids	[2,10,11,12,13,14,15,16]
*Boswellia elongata* Balf.f.	Endemic	Resin; bark	Resin chewed for teeth weakness and gum infection; fever and pain, stomach complaints, asthma (organ not specified)	Resin: mono-, sesqui- and diterpenes; pentacyclic and tirucallane-type triterpenoid acids including boswellic acids, KBA and AKBA; incensole. Bark oil: diterpenes (incensol)	[3,17,18,19,20,21]
*B. socotrana* Balf.f.	Endemic	Resin; bark	Resin for stomatitis; chewed for tooth decay, teeth weakness and gum infection	Resin oil: oxygenated monoterpenes. Bark oil: monoterpenes, aromatic ketone	[3,17,19,22]
*B. ameero* Balf.f.	Endemic	Resin	Resin chewed for teeth weakness and gum infection; fumigant for colds	Resin oil: monoterpenes and aromatic compounds	[3,17]
*B. dioscoridis* Thulin	Endemic	Bark	Not identified at species level	Bark oil: monoterpene hydrocarbons	[19]
Other Socotran *Boswellia* species (*B. asplenifolia*, *B. bullata*, *B. hesperia*, *B. nana*, *B. oropedionis*, *B. popoviana*, *B. samhaensis*, *B. scopulorum*)	Endemic	Fresh resin volatiles for *B. bullata*, *B. nana* and *B. popoviana* only	Resin of *B. nana* and *B. popoviana* chewed for teeth weakness and gum infection; others not identified	Volatile terpenoids	[3,4,23,24]
*Commiphora ornifolia* (Balf.f.) J.B.Gillett	Endemic	Resin; bark	Bark and underbark in *naqf* wound powder; bark (and resin) also as antiseptic, for diarrhoea and dysentery, and as emmenagogue; resin for toothache; resin chewed for mouth sores and tooth decay; bark or resin applied to wounds	Resin: sesquiterpenes, furofuran lignan (yangambin), polysaccharides. Bark oil: oxygenated monoterpenes	[3,25,26,27]
*C. parvifolia* (Balf.f.) Engl.	Endemic	Resin; bark	Resin chewed for mouth sores and tooth decay; genus-level bark use (*naqf*)	Resin: monoterpenes. Bark oil: oxygenated sesquiterpenes, fatty acids	[3,25,26]
*C. socotrana* (Balf.f.) Engl.	Endemic	Resin; leaves	Resin chewed for mouth sores and tooth decay; genus-level bark use (*naqf*)	Resin volatiles: oxygenated monoterpenes. Leaves: chromone glycoside	[3,25,26,28]
*C. planifrons* (Balf.f.) Engl.	Endemic	Resin	Resin chewed for mouth sores and tooth decay; genus-level bark use (*naqf*)	Resin volatiles: monoterpenes	[3,25,26]
*C. kua* (R.Br. ex Royle) Vollesen	Native, not endemic	Resin	Genus-level bark use (*naqf*)	Resin volatiles: sesquiterpenes	[25,26]

Abbreviations: IUCN, International Union for Conservation of Nature; KBA, 11-keto-β-boswellic acid; AKBA, 3-O-acetyl-11-keto-β-boswellic acid.

Earlier reviews have treated dragon’s blood across several source species [6] or focused on *D. cinnabari* alone [29]. A recent review examined the medicinal flora of Socotra as a source of anti-infective agents, with emphasis on antimicrobial resistance, ethnopharmacology, phytochemical diversity and lead optimization [30]. The present review has a distinct, narrower focus. It is restricted to the three traditional resin-producing groups of the archipelago, *D. cinnabari*, Socotran *Boswellia* and Socotran *Commiphora*, and it emphasizes species-specific phytochemistry, the separation of resin evidence from evidence obtained with bark or other organs, biological activities beyond anti-infective endpoints, toxicological evidence, and the limits of extrapolating from non-Socotran taxa. Its objective is to critically appraise the published evidence on the traditional uses, chemical composition, biological activities and safety of these resins, and to state explicitly which conclusions are supported by the evidence.

## 2. Literature Search and Scope

This critical narrative review used a targeted, non-systematic literature search and does not claim exhaustive retrieval. Relevant literature was identified through searches of PubMed, Web of Science, and Google Scholar from database inception through 29 September 2026. Searches were conducted in English using botanical names, accepted synonyms, and variant spellings, supplemented by geographical and topic-specific terms. Geographical terms included Socotra, Soqotra, and Socotra Archipelago. Botanical terms covered *Dracaena cinnabari* and the recognised Socotran *Boswellia* and *Commiphora* species, including the older spelling *B. dioscorides*. Resin-related terms included dragon’s blood, *dam al-akhawain*, frankincense, resin, oleo-gum resin, and essential oil. Additional terms included phytochemistry, bioactive compounds, biological activity, antimicrobial, antifungal, antioxidant, anti-inflammatory, cytotoxic, anticancer, toxicity, safety, traditional use, and ethnomedicine. Individual botanical names and relevant combinations of these terms were used to identify publications across the three databases. The searches were topic-directed rather than conducted using a prespecified systematic-review search algorithm. These three resin groups were selected because their traditional medicinal use is documented in Socotran ethnobotanical literature and published phytochemical or biological evidence is available for at least some constituent species [2,3,4].

Relevant publications were retrieved, downloaded, and examined in full. Articles published online by 29 September 2026, including those published ahead of print, were considered; eligibility was determined by the first online publication date rather than subsequent assignment to a journal issue. Searches were supplemented by backward reference checking of original studies and relevant reviews, and bibliographic information was verified against journal and publisher records.

Peer-reviewed original studies were included when the botanical identity of the Socotran species and the plant organ investigated (resin, bark, or another part) could be established from the original report. Studies lacking sufficient information to establish botanical identity or the tested plant organ were excluded from the evidence synthesis. Studies involving resin-derived compounds from species endemic to Socotra without explicit documentation of the collection locality were retained only as taxon-level contextual evidence and were not considered evidence from provenance-verified Socotran resin. When studies reported multiple plant organs, individual experimental findings were assigned only to the organ explicitly identified as the tested material. A voucher number covering several organs was not considered sufficient evidence that resin had been tested. Findings from bark or other non-resin material were retained as contextual evidence and distinguished from resin-specific findings. Reviews were used for background information and reference tracing rather than as sources of experimental findings when primary studies were available. Conference abstracts were not used as evidence.

The evidence was critically appraised according to botanical identity, plant part, preparation type, experimental methods, and reported outcomes. Phytochemical findings were evaluated by distinguishing isolated compounds from constituents detected through analytical profiling, with attention to sample provenance, quantitative data, and reported analytical validation. Pharmacological evidence was assessed in relation to the tested material, experimental model, assay endpoint, concentration or dose, controls, and selectivity where available. Findings from bark, computational analyses, or non-Socotran taxa were not considered direct evidence of pharmacological activity of Socotran resins. Traditional-use reports, including perceived benefits and safety concerns, were treated as ethnobotanical observations rather than experimental evidence of pharmacological efficacy or toxicity.

Botanical nomenclature and author citations follow Plants of the World Online (POWO) [31]. For *Boswellia*, nomenclature was additionally checked against the genus monograph [32] and the original descriptions of species published subsequently [23,24].

## 3. Dragon’s Blood from *Dracaena cinnabari*

### 3.1. Botanical Source and Traditional Uses

*Dracaena cinnabari* Balf.f. (Asparagaceae) is endemic to Socotra [31]. Resin exudes from the stem, either spontaneously or after wounding; the highest grade is known locally as *emzoloh*, and the Arabic name *dam al-akhawain* (“blood of the two brothers”) is applied to both the tree and the resin [2]. Anatomical work on botanic-garden trees of *D. cinnabari* and two Macaronesian species indicates that resin is also secreted during normal leaf abscission, not only after injury [33].

The most detailed quantitative ethnobotanical data were obtained from a survey of 100 informants in ten localities [2]. All informants reported medicinal use of the resin. Citation frequencies were highest for dermal conditions (100%), dental conditions (100%), eye conditions (98%), internal and external bleeding, including postpartum haemorrhage (95%), and gastrointestinal complaints (94%). The resin was applied topically as a powder, suspension or paste, and taken orally in water or milk [2]. Two limitations affect the interpretation of these data: the interviews were conducted in 1990, although the report was published in 2018, and the survey concerned a single species, so informant consensus cannot be compared with that for other Socotran remedies. The same survey reported that 80% of informants believed the resin could induce abortion [2]. This finding reflects an ethnobotanical perception rather than an observed clinical outcome or experimentally demonstrated reproductive toxicity. The reported percentages reflect the frequency with which informants cited particular uses or beliefs, not the proportion of treatments that were effective. Perceived benefits and traditional safety concerns should therefore be distinguished from experimentally demonstrated biological activities and toxicological findings, which are evaluated separately in Section 3.3 and Section 3.4.

A broader ethnopharmacological survey, based on interviews with 105 informants in 1990, documented 86 endemic medicinal plants on Socotra [3]. Market samples sold as *dam al-akhawain* in Aden and Sana’a were partly or completely substituted, in some cases by *Euclea divinorum* root powder or powdered red coral [2]. Authentication is therefore a precondition for interpreting any biological study of purchased material.

### 3.2. Phytochemistry

The non-volatile fraction of *D. cinnabari* resin is characterized principally by phenolic constituents, including homoisoflavans, flavans, chalcones and dihydrochalcones [11]. Early isolation studies also identified several previously unreported representatives of these classes, while subsequent work expanded the chemical profile to oligomeric flavonoids, including cinnabarone and damalachawin, as well as the metacyclophane dracophane [10,13,14]. Sterol and triterpenoid constituents have also been reported, with lophenol representing the predominant sterol in the analyzed fraction [12]. These studies were conducted using resin collected from Socotra and documented with voucher material [10,11,12,13,14]. Among the flavylium chromophores contributing to the red colour of dragon’s blood, 7,4′-dihydroxyflavylium was identified as a species marker for *D. cinnabari* using high-performance liquid chromatography with diode-array detection (HPLC–DAD). This marker distinguished *D. cinnabari* from *D. draco* (dracoflavylium) and *Daemonorops* species (dracorhodin) in reference samples that included 11 *D. cinnabari* resin samples collected on Socotra. Application of the method to 37 historical samples in the Economic Botany Collection at Kew revealed inconsistencies in sample labelling [15].

**Figure 1:**
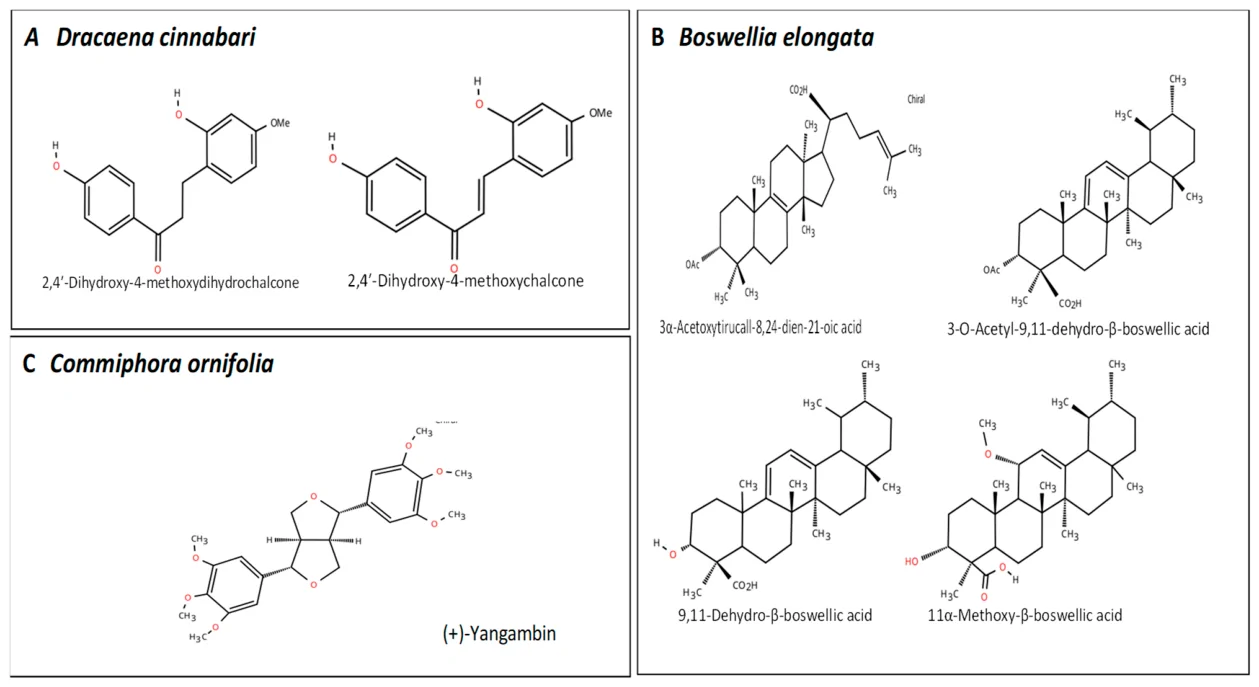
Selected chemical structures of constituents isolated from Socotran medicinal resins. (**A**) Two chalcone derivatives isolated from *Dracaena cinnabari* resin and evaluated for their effects on hepatitis B virus antigen production [34]. (**B**) Four triterpenoid acids isolated from *Boswellia elongata* resin and evaluated for yeast α-glucosidase inhibition [20]. (**C**) (+)-Yangambin isolated from *Commiphora ornifolia* resin, for which no compound-specific biological activity was established in the cited study [27]. Chemical structures were drawn using FreeChemDraw (version 1.0, 2026; https://freechemdraw.com/, accessed on 19 October 2026) based on the structures reported in the original publications.

Two chalcone derivatives were later isolated from the resin by bioassay-guided fractionation [34]. Analysis of the volatile fraction using solid-phase microextraction (SPME) with comprehensive two-dimensional gas chromatography–mass spectrometry (GC–MS) identified 20 terpenoids in the resins of five *Dracaena* taxa; monoterpene profiles differed between species, with α-pinene, camphene, β-pinene and δ-3-carene characteristic of *D. cinnabari* from Socotra, suggesting potential chemotaxonomic markers [16]. In the headspace analysis, trans-muurola-3,5-diene accounted for approximately 44.1% of the total chromatographic peak area in the *D. cinnabari* resin volatile profile [16]. This relative abundance does not represent its concentration in the whole resin. These findings represent different forms of chemical evidence: isolation and structural characterisation of non-volatile constituents [10,11,12,13,14,34], marker-based resin authentication [15], and chromatographic profiling of volatile constituents [16]. Compounds identified or described as predominant within an analysed fraction cannot necessarily be considered major constituents of the whole resin, and chemical identification alone does not establish biological activity. Although HPLC–DAD has been used to distinguish *D. cinnabari* from other dragon’s blood sources through flavylium markers [15], quantitative concentrations of the principal homoisoflavans and chalcones in authenticated resin remain insufficiently documented. This limits comparisons among preparations and the interpretation of pharmacological dose–response relationships. Representative structures of two chalcone derivatives isolated from *D. cinnabari* resin are included in Figure 1.

### 3.3. Experimentally Reported Biological Activities

The biological findings for *D. cinnabari* resin differ in the extent to which they can be attributed to individual constituents. Compound-level testing was identified in only two sets of studies: two chalcone derivatives isolated from the resin were tested for effects on viral antigen production in cell culture [34], and homoisoflavonoids and chalcones derived from the resin were tested in mouse liver microsomes [35]. The collection provenance of the material used in the latter study was not documented, so its results are treated as taxon-level contextual evidence (Section 6). All other activities were measured with whole resin, solvent extracts or fractions, so the responsible constituents remain unidentified. Each subsection therefore states which constituents, if any, can be linked to the reported activity. Structure–activity relationships (SARs) are discussed only where two or more tested compounds allow a comparison, and these comparisons are exploratory.

#### 3.3.1. Antimicrobial and Antifungal Activity

A methanolic solution of authenticated resin produced inhibition zones of 20–30 mm against *Aspergillus fumigatus*, *Microsporum gypseum* and *Trichophyton mentagrophytes* in disc diffusion (2 mg per disc), comparable to nystatin (100 µg per disc); its minimum inhibitory concentration (MIC) against the two dermatophytes was 500 µg/mL [2]. Chloroform and methanol extracts of the resin (4 mg per disc) produced inhibition zones of 10–22 mm, including the 6 mm disc, against Gram-positive bacteria, including multiresistant staphylococci, and the chloroform extract also produced a 12 mm zone against *Escherichia coli*; extracts of the leaves were inactive [36]. An ethanolic extract of Socotran resin produced inhibition zones of 4.9–11.5 mm against Gram-positive and Gram-negative bacteria and fungi in disc diffusion [37]. That report is internally inconsistent: zone diameters were stated to include the 6 mm disc, yet values below 6 mm were given, and the reported MICs (1.25–2.5 µg/mL) are several hundred-fold lower than the MIC of 500 µg/mL measured for authenticated resin [2]; its MIC values are therefore not used here. These findings represent screening data; no study using an infection model was identified. None of these studies identified the constituents responsible for the antimicrobial or antifungal effects, so no compound-specific SAR can be derived.

#### 3.3.2. Antioxidant Activity

The methanolic solution of whole resin scavenged 2,2-diphenyl-1-picrylhydrazyl (DPPH) radicals with a half-maximal inhibitory concentration (IC_50_) of 25.5 µg/mL, compared with 15.5 µg/mL for ascorbic acid [2]. Among homoisoflavonoids and chalcones isolated from the resin, a homoisoflavan inhibited Fe^2+^/reduced nicotinamide adenine dinucleotide phosphate (NADPH)-dependent lipid peroxidation in mouse liver microsomes with a potency comparable to that of quercetin, whereas the chalcones tested were poor antioxidants [35]. However, the original study identifies the active homoisoflavan inconsistently as H2 in the Abstract and H1 in the Discussion, preventing unambiguous compound-specific attribution. The contrast between the homoisoflavan and the chalcones compares two different scaffolds and is therefore not a structure–activity relationship within a compound class, and the unresolved H1/H2 labelling prevents relating the activity to a specific substitution pattern. The constituents responsible for the radical-scavenging activity of the whole resin were not identified [2].

#### 3.3.3. Cell-Based Viral Assays

In a hepatitis B virus (HBV) cell-based assay, a methanol extract of Socotran resin reduced hepatitis B surface antigen (HBsAg) production in HepG2.2.15 cells (IC_50_ 20.93 µg/mL) and showed a 50% cytotoxic concentration (CC_50_) of 288.24 µg/mL in the same cells, corresponding to a therapeutic index (TI; CC_50_/IC_50_) of 13.8 [38]. Bioassay-guided fractionation of a resin methanol extract yielded two chalcone derivatives, 2,4′-dihydroxy-4-methoxydihydrochalcone and 2,4′-dihydroxy-4-methoxyhydrochalcone, which reduced HBsAg production with IC_50_ values of 20.56 and 6.36 µg/mL, respectively (CC_50_ 346.7 and 242.1 µg/mL; therapeutic indices 16.9 and 38.1) [34].

The two isolated derivatives share the same aromatic substitution pattern but differ in their α,β-carbon linkage: 2,4′-dihydroxy-4-methoxydihydrochalcone contains a saturated chain, whereas 2,4′-dihydroxy-4-methoxychalcone has an (E)-α,β-double bond. Although the latter was designated 2,4′-dihydroxy-4-methoxyhydrochalcone in the original publication [34], its molecular formula (C_16_H_14_O_4_) and two olefinic proton signals (δ 7.52 and 7.57, J = 15.7 Hz) support the α,β-unsaturated chalcone structure shown in Figure 1. 2,4′-Dihydroxy-4-methoxychalcone showed a lower IC_50_ for reducing HBsAg production than 2,4′-dihydroxy-4-methoxydihydrochalcone (6.36 versus 20.56 µg/mL). The molecular weights of the two compounds are almost identical (about 270 and 272 g/mol), so the difference is similar on a molar basis. The unsaturated compound was also somewhat more cytotoxic (CC_50_ 242.1 versus 346.7 µg/mL), but its greater potency gave a higher therapeutic index (38.1 versus 16.9) [34]. Although this difference suggests a possible influence of α,β-unsaturation on the observed activity, comparison of only two compounds is insufficient to establish a general structure–activity relationship or identify the structural basis of the effect. A mechanistic role of the α,β-unsaturated carbonyl has not been demonstrated.

The two compounds were isolated in markedly different amounts (90 mg and 4 mg from 3 g of extract), and no in vivo data were reported. The markedly different isolation yields do not establish the relative contributions of these compounds to the HBsAg-reducing activity of the crude extract, which would require quantitative analysis and activity-guided confirmation. Both HBV studies assessed HBsAg and hepatitis B e antigen (HBeAg) production in HepG2.2.15 cell cultures [34,38]. Although HBeAg is associated with viral replication, neither study directly quantified HBV DNA. The observed reductions in viral antigen production therefore represent preliminary in vitro findings and do not establish inhibition of HBV replication or therapeutic efficacy in vivo. An earlier screen in which a methanol extract of *D. cinnabari* inhibited influenza A virus and herpes simplex virus type 1 in cell culture [39] has been cited as evidence for antiviral activity of the resin.

However, the voucher number given (SP-D225) covers leaves, flowers and resin, which were extracted and reported separately in companion studies [36,40,41], and the antiviral screen lists only leaves and flowers as the material tested [39]. Its result is therefore not attributed to the resin here.

#### 3.3.4. Antiprotozoal Activity

A methanol extract of the resin (voucher SP-D225) inhibited *Plasmodium falciparum* (IC_50_ 2.1 µg/mL), *Trypanosoma cruzi* (4.1 µg/mL), *T. brucei* (8.0 µg/mL) and *Leishmania infantum* (8.1 µg/mL), but was similarly cytotoxic to MRC-5 human lung fibroblasts (IC_50_ 7.7 µg/mL), so the activity was judged non-selective [41]. The constituents responsible were not identified, and no structure–activity relationship can be inferred.

#### 3.3.5. Cytotoxic and Antitumour Activity

A methanol extract of authenticated Socotran resin was cytotoxic to six oral squamous cell carcinoma (OSCC) cell lines (IC_50_ 5.9–19.2 µg/mL after 72 h), most markedly to H400 cells, whereas normal human oral fibroblasts were not inhibited at the highest concentration tested (30 µg/mL). In H400 cells, the extract and its most active fractions induced apoptosis with mitochondrial membrane depolarisation, activation of caspase-9 and caspase-3/7 and accumulation of cells in S phase, and a semiquantitative protein array showed increased Bax, Bad and Bid and decreased Bcl-2; the change in cytochrome *c* was small and not statistically significant [42]. In H103 tongue carcinoma cells, a resin methanol extract (IC_50_ 5.5 µg/mL) inhibited proliferation and migration, caused S- and G2/M-phase arrest and induced apoptosis through the intrinsic pathway, while normal human oral fibroblasts were not inhibited at 30 µg/mL [43]. The IC_50_ values reported in that study for three further OSCC lines (H314, H357 and H376) are identical to those reported earlier [42]. In male rats given 4-nitroquinoline-1-oxide (4NQO) in drinking water for 8 weeks, oral administration of the resin methanol extract at 100, 500 or 1000 mg/kg, from 1 week before to 1 week after carcinogen exposure, was associated with a lower incidence of histologically confirmed OSCC at 22 weeks (42.9%, 28.6% and 14.3%, respectively, versus 85.7% in the carcinogen-only group; seven rats per group) and with smaller tongue tumors at 500 and 1000 mg/kg. At 1000 mg/kg, immunohistochemistry (three samples per group) showed reduced cyclin D1, Ki-67, Bcl-2 and p53 staining [44]. In a second protocol, oral administration of the extract at the same doses for 10 weeks, starting 1 week after the end of an 8-week 4NQO exposure, was associated with OSCC incidences at 22 weeks of 57.1%, 28.6% and 14.3%, respectively, versus 85.7% in the carcinogen-only and vehicle groups (seven rats per group) [43]. These data come from one research group, a single carcinogen model, small groups and crude extracts; neither protocol tested treatment of established, measured tumors, and the active constituents have not been identified. Selectivity for cancer cells has varied across studies. The methanolic solution of resin showed low cytotoxicity against FL cells (a human amniotic epithelial cell line) (IC_50_ 300 µg/mL) [2], whereas a resin methanol extract was cytotoxic to MRC-5 fibroblasts at 7.7 µg/mL [41]. In a further study of resin stated to originate from Socotra, a methanol extract was less cytotoxic to A549 lung cancer cells than to non-cancerous HEK-293 cells after 24 h (IC_50_ 90.96 versus 72.10 µg/mL), indicating a lack of selectivity for cancer cells [45]. A combined extract of Socotran resin and *Aloe perryi* flowers was cytotoxic to HT-29 colorectal cancer cells (IC_50_ 28.56 µg/mL), but the design does not isolate the contribution of the resin [46]. Beyond the two chalcone derivatives evaluated for reductions in HBsAg production [34], the available antimicrobial, antifungal, antiprotozoal, and anticancer-related studies of *D. cinnabari* resin preparations did not identify the individual constituents responsible for the observed effects. Consequently, reliable compound-specific structure–activity relationships cannot be established for these endpoints from the available evidence. Direct comparisons of IC_50_ values and cancer-cell selectivity across these studies are limited by differences in the cell lines examined, exposure durations, cytotoxicity assay methods, and extract preparations.

#### 3.3.6. Compound-Specific Evidence and SAR Limitations

A separate enzyme-assay comparison from the isolated-compound study [35] concerns cytochrome P450 1A (CYP1A)-dependent 7-ethoxyresorufin O-deethylase (EROD) activity in mouse liver microsomes. Two isolated chalcones, 2-hydroxychalcone and 4,6-dihydroxychalcone, inhibited EROD, the former more strongly [35]. The compounds differ in both the number and position of their hydroxyl groups, but this limited comparison does not establish the contribution of individual substitutions to enzyme inhibition. This endpoint is distinct from antioxidant activity, and its toxicological relevance is considered in Section 8.

Across the biological endpoints reviewed, compound-level activity was reported in only two sets of studies: the chalcone derivatives evaluated for their effects on HBsAg production [34] and the homoisoflavan and chalcones tested in mouse liver microsomes [35]. The available structural comparisons are limited to the saturated and α,β-unsaturated chalcone pair and two chalcones with different hydroxylation patterns evaluated for EROD inhibition. Neither comparison is sufficient to establish a general structure–activity relationship. The antimicrobial, antifungal, antiprotozoal, and anticancer studies used resin preparations without identifying the individual constituents responsible for their observed effects. Consequently, compound-specific SAR cannot be reliably established for these activities. Other constituents isolated from the resin, including cinnabarone, damalachawin, and dracophane [10,13,14], have not been experimentally linked to the biological activities discussed here.

### 3.4. Safety and Toxicity

The only formal toxicity study identified used *D. cinnabari* resin collected on Socotra and authenticated with a deposited voucher specimen. The study followed Organisation for Economic Co-operation and Development (OECD) Test Guidelines 423 and 407 for acute and 28-day repeated-dose oral toxicity, respectively, with slight modifications. Extraction of 50 g of powdered resin with methanol yielded 28.0 g of dry extract (56% yield, calculated from the reported masses). The extract was dissolved in 10% dimethyl sulfoxide (DMSO) and administered by oral gavage at 10 mL/kg body weight. All reported doses refer to the dry methanolic extract rather than the unextracted resin. In the acute toxicity test, nine non-pregnant, nulliparous female Sprague–Dawley rats were divided into three groups (*n* = 3 per group: vehicle control, 300 mg/kg, and 2000 mg/kg). Neither extract dose caused mortality or observable signs of toxicity during the 14-day observation period. Based on these findings, the authors classified the extract as Category 5 under the Globally Harmonized System of Classification and Labelling of Chemicals (GHS).

In the 28-day repeated-dose study, 60 rats (30 males and 30 females) were allocated to six groups (*n* = 10 per group, five per sex): one vehicle control group, three extract-treated groups receiving 500, 1000, or 1500 mg/kg daily, and two satellite groups receiving vehicle or 1500 mg/kg, respectively. The satellite groups were observed for an additional 14 days after treatment cessation. No significant treatment-related differences were reported in body weight, food consumption, organ weights, haematological parameters, serum biochemistry, or histopathological findings in the liver, kidney, heart, spleen, and lungs. However, water intake was significantly higher in the extract-treated groups than in the controls, including in the high-dose satellite group, and the cause of this increase was not established [47]. No study of reproductive or developmental toxicity, genotoxicity, chronic exposure, or topical and ocular application, the main routes of traditional use, was identified. The reported belief that the resin could induce abortion comes from ethnobotanical interviews [2] and does not constitute experimental evidence of reproductive toxicity. An early study applied an electrochemical prescreening index of “potential carcinogenicity” (direct-current polarography in the presence of α-lipoic acid) to seven phenolics isolated from the resin: most gave low index values, comparable with those of weakly or non-carcinogenic polycyclic aromatic hydrocarbons, whereas one homoisoflavan gave a higher value [48]. This index is not a validated biological test, and no genotoxicity or carcinogenicity study in biological systems was identified.

### 3.5. Evidence Limitations

The evidence for *D. cinnabari* consists of detailed structural chemistry, one quantitative ethnobotanical survey, in vitro screening studies, a coherent set of preclinical oral cancer studies from one group, and one rodent toxicity study. No eligible human study was identified in the literature searched. Activities frequently attributed to dragon’s blood in general, including wound healing, haemostasis and antiulcer effects, were not supported by any study of authenticated Socotran resin that met the inclusion criteria of this review.

## 4. Frankincense from Socotran *Boswellia* Species

### 4.1. Socotran Species

The monograph of the genus recognized 11 *Boswellia* species in the Socotra Archipelago, one of which, *B.* “*hesperia*”, was proposed only provisionally because no specimens were then available [32]. *Boswellia hesperia* Thulin, M.H.Weber & Rzepecky, a dwarf tree restricted to westernmost Socotra, was formally described in 2025 and assessed by the describing authors as Critically Endangered under IUCN criteria [23]; because it had earlier been depicted as a dwarf form of *B. popoviana* [23], older records under that name require careful interpretation. The subsequent description of *B. oropedionis* Thulin & M.H.Weber, known from a single population of approximately five trees on the Qatariyah plateau in southern Socotra and preliminarily assessed as Critically Endangered, brought the number of endemic *Boswellia* species in the archipelago to 12 [24]. These are *B. ameero* Balf.f., *B. asplenifolia* (Balf.f.) Thulin, *B. bullata* Thulin, *B. dioscoridis* Thulin, *B. elongata* Balf.f., *B. hesperia*, *B. nana* Hepper, *B. oropedionis*, *B. popoviana* Hepper, *B. samhaensis* Thulin & Scholte, *B. scopulorum* Thulin and *B. socotrana* Balf.f. [23,24,31,32]. Older studies may use superseded names such as “*B. dioscorides*”. Biological data exist for only four species (*B. ameero*, *B. dioscoridis*, *B. elongata* and *B. socotrana*), and no chemical or biological study of *B. hesperia* or *B. oropedionis* was identified; for *B. bullata*, *B. nana* and *B. popoviana*, and for an unnamed taxon (*B.* sp. A), the only published chemistry is a volatile profile of fresh resin, and no chemical study of *B. asplenifolia*, *B. samhaensis* or *B. scopulorum* was identified [4].

The identity of the plant part studied is a recurring limitation. One set of essential oils of *B. dioscoridis*, *B. elongata* and *B. socotrana* was distilled from the bark, not the oleo-gum resin [19], and the antimicrobial, antiviral and anti-inflammatory studies of *B. elongata* also used bark extracts [18,36,39]. Bark data are reported here because they concern Socotran species, but they do not constitute evidence of frankincense resin activity.

### 4.2. Traditional Uses

The oleo-gum resin of Socotran *Boswellia* is used to relieve pain in dental cavities, to sweeten the breath and to soothe a disturbed stomach [17]. *Boswellia elongata* is reported to be used for fever and pain, to soothe a disturbed stomach and as a remedy for asthma [18], and the resin of *B. socotrana* for stomatitis [22]. Species-level use reports come from an ethnopharmacological survey conducted in 1990: the stem resins of *B. ameero*, *B. elongata*, *B. nana*, *B. popoviana* and *B. socotrana* were chewed for teeth weakness and gum infection and were also used for general weakness, and *B. socotrana* resin was chewed for tooth decay; *B. ameero* resin was used as a fumigant for colds, and the bark of several species was applied as a paste to boils and wounds [3]. Other published accounts often do not state whether a given use refers to the resin, the bark or another organ.

### 4.3. Volatile Chemistry

Published volatile profiles of Socotran *Boswellia* differ markedly between studies, even for the same species. Hydrodistilled resin oils analyzed by GC–MS were dominated by (*E*)-2,3-epoxycarene (51.8%) in *B. socotrana*, by (3*E*,5*E*)-2,6-dimethyl-1,3,5,7-octatetraene (34.9%) and aromatic compounds in *B. ameero*, and by verticiol (52.4%) and caryophyllene (39.1%) in *B. elongata* [17]. The reported essential-oil yields were 1.2%, 1.8%, and 2.3% of dry oleo-gum resin for *B. socotrana*, *B. ameero*, and *B. elongata*, respectively [17]. The percentages reported for individual volatile constituents represent relative GC peak areas within the distilled oils, rather than concentrations in the original resin. Hydrodistilled bark oils had different profiles: α-thujene (9.3%) and α-pinene (8.3%) in *B. dioscoridis*, incensol (14.8%) within a 31.7% diterpene fraction in *B. elongata*, and 2-hydroxy-5-methoxyacetophenone (16.3%), *p*-cymene (13.0%) and camphor (11.6%) in *B. socotrana* [19].

Two later studies analyzed fresh resin collected in the field. GC–MS analysis of methanol extracts of freshly collected resin of Socotran Burseraceae (one to four trees per taxon; trees documented by photographs rather than herbarium vouchers) detected 103 volatiles, of which 53 were fully identified [4]. Thermal desorption GC–MS of *B. elongata* resin from six sites identified 92 terpenoids, with α-pinene, γ-terpinene, *o*-cymene and (*E*)-β-farnesene predominating; composition correlated with temperature, elevation and proximity to the sea, and the differences between sites were subtle and most pronounced in the most geographically isolated population [21].

Differences among these studies may reflect several factors: plant part, sample age, extraction method (hydrodistillation versus thermal desorption), geographic origin and compound identification. The resin-oil study reported only three to seven constituents per oil and used mass-spectral comparisons and retention indices for compound identification [17]. Given the unusual identities and high reported proportions of several constituents, independent confirmation using authentic standards and additional samples would strengthen confidence in these chemical profiles. Until a reproducible profile is established for each species, individual volatiles should not be treated as species markers or as the basis of reported activities.

### 4.4. Non-Volatile Chemistry

The only isolation study identified for a Socotran frankincense resin reported 21 compounds from voucher-documented *B. elongata* resin, demonstrating a chemically diverse non-volatile fraction dominated by triterpenoid constituents [20]. Importantly, the isolated compounds included α- and β-boswellic acids, their acetylated derivatives, and the pharmacologically prominent 11-keto-β-boswellic acid (KBA) and 3-O-acetyl-11-keto-β-boswellic acid (AKBA), together with tirucallane-type triterpenoids and other minor constituents [20]. At least one Socotran species therefore contains the triterpenoid acids best known from *B. serrata* and *B. sacra*. However, no study quantifying boswellic acids in a Socotran species was identified.

### 4.5. Reported Biological Activities by Species

**Resin-derived biological evidence.** The resin essential oil of *B. elongata* showed DPPH radical-scavenging activity (IC_50_ 211.2 µg/mL) and acetylcholinesterase inhibition (29.6% at 200 µg/mL) [17]. Four triterpenoid acids isolated from its resin inhibited yeast α-glucosidase non-competitively (IC_50_ 9.9–56.8 µM) [20]. Molecular docking in the same study was used to explore possible binding at predicted allosteric sites, but these computational findings do not constitute independent experimental evidence of enzyme inhibition [20]. These activity values should not be attributed specifically to KBA or AKBA. Although the tested compounds had lower IC_50_ values than acarbose (942 µM) in the same assay, the findings do not establish antidiabetic efficacy because neither mammalian enzyme activity nor in vivo effects were investigated.

A preliminary structure–activity comparison can be made among the tested triterpenoid acids. 3-O-Acetyl-9,11-dehydro-β-boswellic acid inhibited yeast α-glucosidase more strongly than its non-acetylated analogue, 9,11-dehydro-β-boswellic acid, with IC_50_ values of 9.9 and 20.9 µM, respectively [20]. This difference suggests that acetylation at C-3 may influence inhibitory potency within this structural pair. 11α-Methoxy-β-boswellic acid and the structurally distinct tirucallane-type acid 3α-acetoxytirucall-8,24-dien-21-oic acid also inhibited the enzyme, with IC_50_ values of 14.9 and 56.8 µM, respectively. However, these limited comparisons establish neither the independent contributions of individual functional groups nor a validated molecular mechanism of inhibition.

The resin essential oils of *B. ameero* and *B. socotrana* also showed DPPH scavenging (IC_50_ 175.2 and 121.4 µg/mL, respectively) and acetylcholinesterase inhibition (41.6% and 59.3%, respectively, at 200 µg/mL) [17]. Aqueous, methanol, and chloroform extracts of authenticated *B. socotrana* resin exhibited weak cytotoxicity toward non-malignant FL cells (IC_50_ 454.6–535.1 µg/mL) [22].

**Bark-derived biological evidence (contextual findings).** Methanol extracts of *B. elongata* and *B. ameero* bark inhibited Gram-positive bacteria, including multidrug-resistant *Staphylococcus* isolates, in agar diffusion assays [36]. In cell culture, their bark extracts inhibited influenza A virus and herpes simplex virus type 1, with IC_50_ values of 3.1 and 0.35 µg/mL for *B. elongata* and 12.5 and 1.5 µg/mL for *B. ameero*, respectively [39]. In rodent models, *B. elongata* bark extract administered orally at 400 mg/kg reduced carrageenan-induced paw oedema by 82%, cotton-pellet granuloma weight by 51%, and acetic acid-induced writhing by 67% [18]. Neither *B. elongata* nor *B. ameero* bark extracts inhibited the five human cancer cell lines tested (IC_50_ > 50 µg/mL) [40]. Bark essential oils exhibited comparatively weak antibacterial and antioxidant effects [19].

For *B. socotrana*, bark methanol extracts inhibited *Staphylococcus aureus* and *Micrococcus flavus* (MIC 125 µg/mL) and showed cytotoxicity against selected cancer cell lines, although selectivity over non-malignant cells was not established [49]. The same bark material inhibited *Trypanosoma cruzi* and *T. brucei* (IC_50_ 8.3 and 9.3 µg/mL; selectivity indices 3.9 and 3.5, respectively) [41] and reduced HBsAg production in HepG2.2.15 cells (IC_50_ 118.9 µg/mL; therapeutic index 2.4) [38]. For *B. dioscoridis*, all available biological findings concern bark: extracts showed antibacterial activity (MIC 125 µg/mL against *S. aureus* and *M. flavus*), radical-scavenging and cytotoxic effects [49], and reduced HBsAg production in HepG2.2.15 cells (IC_50_ 24.5 µg/mL; therapeutic index 14.8) [38].

These bark-derived findings provide context for the investigated Socotran species but do not establish biological activity of their resins. Additional assay results are presented in Table 2.

### 4.6. Evidence Limitations

For Socotran frankincense resin, the biological evidence is limited to in vitro assays of essential oils, crude extracts and isolated compounds. The only animal data identified concerned a bark extract of *B. elongata*. No toxicity study of a Socotran *Boswellia* resin and no eligible human study were identified in the literature searched. Findings from the anti-inflammatory and clinical literature on boswellic acid-standardised extracts of *B. serrata* and *B. sacra* cannot be extrapolated to Socotran species, whose boswellic acid content has not been measured.

## 5. Resins from Socotran *Commiphora* Species

### 5.1. Socotran Species

Five *Commiphora* species occur on Socotra: *C. kua* (R.Br. ex Royle) Vollesen, which is widespread from Eritrea to Zambia and the southern Arabian Peninsula, and the endemic species *C. ornifolia* (Balf.f.) J.B.Gillett, *C. parvifolia* (Balf.f.) Engl., *C. planifrons* (Balf.f.) Engl. and *C. socotrana* (Balf.f.) Engl. [25,31]. None of these is *C. myrrha*, and pharmacological data obtained with *C. myrrha* cannot be assumed to apply to them.

### 5.2. Traditional Uses

*Commiphora* species are among the most important medicinal plants in Socotran medicine, but the principal documented remedy uses bark rather than resin. The bark and underbark are used to make *naqf*, a powdered dressing applied to sores, ulcers and wounds in people and livestock [25]. For *C. ornifolia*, both the bark and the aromatic resin are reported to be used, including antiseptic use, treatment of diarrhoea and dysentery, and use as an emmenagogue, although the organ associated with each use is not always specified [27]. The resin of *C. ornifolia* is also inserted into tooth cavities to relieve toothache [27]. In a 1990 ethnopharmacological survey, the dried resins of all four endemic species were chewed or eaten for mouth sores, intestinal worms, nausea and tooth decay; preparations of *C. ornifolia* bark or resin were applied to wounds, including circumcision wounds, a use also recorded for the other species, although the organ to which it is attributed differs between the tables of that survey [3].

### 5.3. Resin Chemistry and Major Constituents

Chemical knowledge of Socotran *Commiphora* resins remains limited and fragmentary. An earlier phytochemical study of *C. socotrana* concerned leaves, not resin, and yielded a new chromone glycoside, 7-*O*-methylaloeresin A [28]. Analysis of a single fresh resin sample of each Socotran *Commiphora* species detected phytol and ledene, which had not previously been reported from *Commiphora* [4]. A headspace SPME–GC–MS study of resins from five species, with two accessions each, found sesquiterpenes predominant in *C. kua* and partly in *C. ornifolia*, monoterpenes in *C. planifrons* and *C. parvifolia*, and oxygenated monoterpenes in *C. socotrana*; the dominant compounds were γ-cadinene in *C. kua*, β-eudesmol or δ-cadinene in the two *C. ornifolia* accessions, limonene in *C. planifrons* and *C. parvifolia*, and terpinen-4-ol in *C. socotrana* [26]. Headspace sampling captures the most volatile fraction and is not directly comparable with hydrodistillation data.

The first study of the non-volatile fraction of a Socotran *Commiphora* resin isolated the furofuran lignan (+)-yangambin from *C. ornifolia* resin. HPLC–DAD analysis quantified yangambin in all eight resin samples at 3.50 ± 0.02% to 9.05 ± 0.19% (*w*/*w*), expressed as the mass of yangambin relative to the total recovered ethanol extract rather than the starting dry resin or an isolated fraction [27]. For the Killesan sample (RC7), the reported ethanol extraction yield was 13% of the starting resin mass; this single-sample yield should not be generalized to other populations. The analytical method reported a calibration coefficient of R^2^ = 0.999, a detection limit of 77.9 ng, and a quantification limit of 236 ng [27]. The variation in yangambin content among samples demonstrates chemical variability but does not establish distinct chemotypes. Analysis of the hydrolysed polysaccharide fraction identified arabinose, rhamnose, galactose and galacturonic acid [27]. Biological activities reported for yangambin from other plant sources have not been tested with material from *C. ornifolia*.

Hydrodistilled bark oils have a different composition. The oil of *C. ornifolia* bark was dominated by camphor (27.3%) and α-fenchol (15.5%), and that of *C. parvifolia* bark by hexadecanoic acid (18.4%), caryophyllene oxide (14.2%) and β-eudesmol (7.7%) [25].

### 5.4. Reported Biological Activities

**Bark-derived biological evidence.** No eligible biological study of Socotran *Commiphora* resin was identified; the available experimental findings involve bark extracts or bark essential oils. In broth microdilution assays, the essential oils of *C. ornifolia* and *C. parvifolia* inhibited mainly Gram-positive bacteria, with MIC values of 0.40–3.25 and 2.18–8.75 mg/mL, respectively, but showed weak DPPH radical-scavenging activity [25]. Bark methanol extracts also showed antibacterial effects against Gram-positive organisms [36,49]. A *C. parvifolia* bark extract inhibited herpes simplex virus type 1 in cell culture (IC_50_ 13.0 µg/mL), but not influenza A virus [39], and showed limited antiproliferative activity against the cancer cell lines examined [40]. Extracts of *C. ornifolia* bark exhibited antibacterial, radical-scavenging, and antiproliferative effects [49], whereas no HBsAg inhibition was reported in an HBV antigen assay [38]. A chloroform extract of *C. ornifolia* bark was cytotoxic to non-malignant FL cells (IC_50_ 39.3 µg/mL), while aqueous and methanol extracts showed substantially lower cytotoxicity (IC_50_ > 450 µg/mL) [22]. These findings provide contextual information about the investigated species but cannot be extrapolated to their resins. Detailed assay results are provided in Table 2.

### 5.5. Evidence Limitations

For Socotran *Commiphora* resins, the evidence consists of volatile profiles and one non-volatile isolation study. No biological activity, toxicity, or human study involving these resins was identified in the literature searched. The pharmacology of *C. myrrha* resin cannot be transferred to Socotran species, whose known chemical profiles differ from it.

## 6. Phytochemical Constituents and Evidence of Biological Activity

The resin groups differ in reported chemistry (Table 3). *Dracaena cinnabari* resin contains homoisoflavans, flavans, chalcones and dihydrochalcones, the biflavonoid cinnabarone, the triflavonoid damalachawin and the metacyclophane dracophane; sterols and a volatile terpenoid fraction are also reported [10,11,12,13,14,16]. Socotran *Boswellia* and *Commiphora* produce oleo-gum resins with resinous, gum and volatile-oil fractions. Studies have focused largely on their volatile fractions and have identified monoterpenes, sesquiterpenes and diterpenes [4,17,21,26]. *B. elongata* resin also yielded non-volatile pentacyclic and tirucallane-type triterpenoid acids, including boswellic acids and AKBA [20]. In *Commiphora* resins, the only non-volatile constituents identified so far are a furofuran lignan and polysaccharides in *C. ornifolia* [27]; the chromone glycoside reported from *C. socotrana* was isolated from leaves [28]. Descriptions of compounds as predominant or minor in individual extracts, distilled oils, or chromatographic profiles should not be extrapolated to their abundance in the whole resin. Although (+)-yangambin has been quantified in recovered ethanol extracts of *C. ornifolia* [27], validated concentration data remain insufficient to classify most individual compounds reliably as major or trace constituents of whole Socotran resins. Biological testing of isolated constituents was identified in three sets of studies: chalcones from *D. cinnabari* resin evaluated for reductions in HBsAg production in HepG2.2.15 cells [34]; homoisoflavonoids and chalcones derived from *D. cinnabari* resin tested for inhibition of mouse liver microsomal lipid peroxidation and cytochrome P450 1A (CYP1A)-dependent 7-ethoxyresorufin O-deethylase (EROD) activity [35]; and triterpenoid acids from *B. elongata* resin tested against yeast α-glucosidase [20]. However, the collection provenance of the material used by Machala et al. [35] was not documented; those findings are therefore treated as taxon-level contextual evidence rather than evidence from provenance-verified Socotran resin.

Selected chemical structures of constituents isolated from the three Socotran medicinal resin groups are presented in Figure 1. These compounds were selected to illustrate structural diversity and the available compound-specific evidence, rather than their quantitative predominance in whole resins [20,27,34].

In contrast, several other constituents, including cinnabarone, damalachawin, dracophane and (+)-yangambin, have been chemically identified in Socotran resins, but no biological assays of these isolated Socotran-derived compounds were identified in the studies reviewed [10,13,14,27]. Their identification should therefore not be interpreted as evidence of demonstrated biological activity. All other activities reviewed here were measured with crude extracts or essential oils, so the responsible constituents are unknown. The presence of a compound class with well-studied pharmacology elsewhere, such as boswellic acids, does not by itself establish that a Socotran resin shares that pharmacology, because activity depends on concentration, on the other constituents present and, in vivo, on absorption, none of which has been measured for Socotran material.

**Figure 2:**
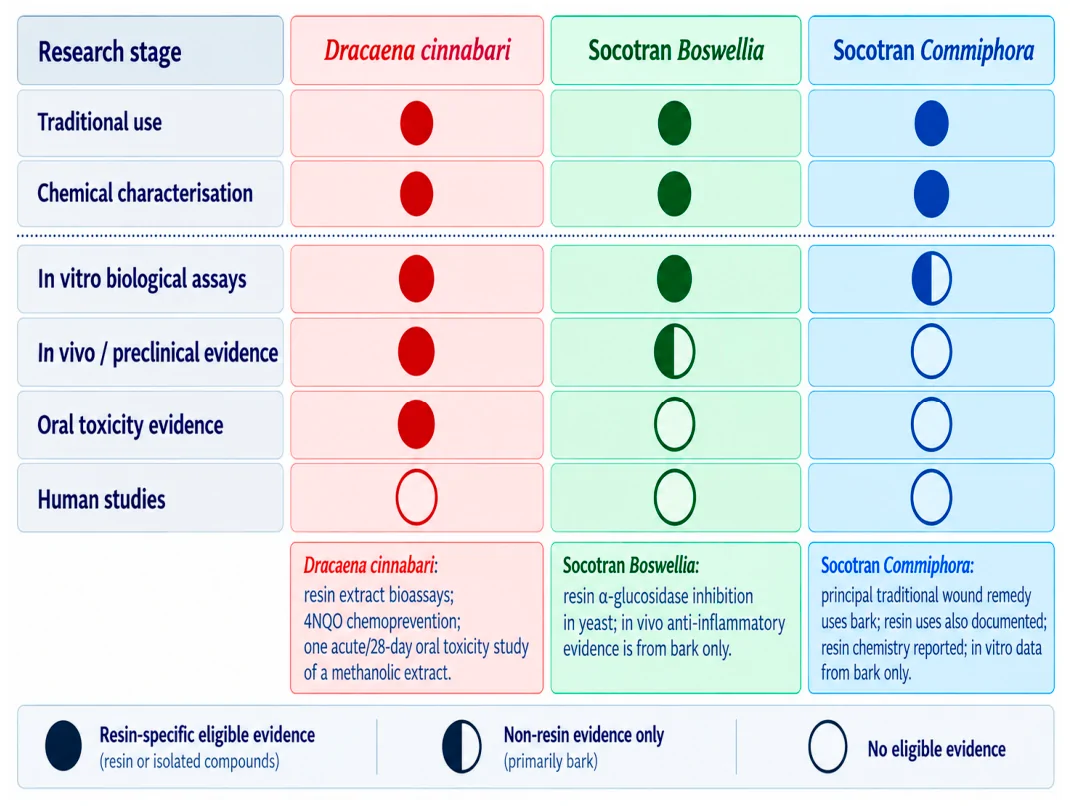
Comparative overview of the evidence for the three traditional medicinal resin groups of Socotra. The figure distinguishes resin-specific findings from contextual evidence derived from bark or other non-resin material across traditional use, chemical characterization, in vitro assays, in vivo studies, oral toxicity, and human studies. Filled circles indicate resin-specific evidence; half-filled circles indicate evidence derived only from non-resin material; and open circles indicate no eligible evidence. Symbols indicate the presence or absence of eligible evidence, not its strength or reproducibility. Traditional use and chemical characterization do not constitute experimental evidence of biological activity. The in vivo anti-inflammatory findings for Socotran *Boswellia* concern bark, whereas the preclinical oral-carcinogenesis studies of *D. cinnabari* used resin extracts from one research group and one carcinogenesis model. Oral toxicity evidence for *D. cinnabari* is limited to a single study involving acute and 28-day administration of a methanolic resin extract. No eligible human studies were identified, and therapeutic efficacy has not been established.

An integrated overview of the three Socotran medicinal resin groups and the progression of evidence across major research stages is presented in Figure 2.

## 7. Comparative Synthesis of Biological Evidence

This section compares the three resin groups by type of evidence; individual results are presented in Section 3, Section 4 and Section 5 and Table 2.

**Traditional use versus experimental evidence.** The most frequently cited traditional indications, namely wound care, bleeding and gastrointestinal complaints, are also the least studied experimentally. For *Commiphora*, the principal wound remedy is prepared from bark rather than resin. No eligible study of these indications with authenticated Socotran resin was identified.

**Antimicrobial activity.** Direct antimicrobial testing of authenticated Socotran resin preparations is concentrated in *D. cinnabari*. A methanolic solution of the resin inhibited dermatophytes (MIC 500 µg/mL), while other resin extracts produced inhibition zones against several bacterial species, particularly Gram-positive isolates [2,36]. However, these findings involve different extraction solvents, applied doses, test organisms, and assay conditions. Agar diffusion results are influenced by the diffusion properties of the tested material and cannot be used for direct comparisons of antimicrobial potency across preparations. The exceptionally low MIC values reported for an ethanolic resin extract [37] require independent confirmation, particularly because some reported inhibition-zone diameters were smaller than the 6 mm discs used. For Socotran *Boswellia* and *Commiphora*, the reported antimicrobial findings concern bark extracts or bark essential oils rather than resin [19,25,36,49]. Antimicrobial efficacy of their resins therefore remains unestablished. Moreover, no eligible infection-model or clinical study was identified for any of the three resin groups.

**Antioxidant activity.** Most evidence derives from chemical radical-scavenging assays; one study also examined Fe^2+^/NADPH-dependent lipid peroxidation in mouse liver microsomes [35]. The methanolic solution of *D. cinnabari* resin was considerably more active than the *Boswellia* essential oils, a pattern consistent with phenolic rather than terpenoid content. Radical scavenging in vitro does not establish antioxidant effects in cells or organisms.

**Anti-inflammatory activity.** The only animal data identified for Socotran *Boswellia* concern a bark extract of *B. elongata*; no anti-inflammatory study of authenticated Socotran resin of any of the three groups met the inclusion criteria. The anti-inflammatory activity of boswellic acids established with *B. serrata* and *B. sacra* cannot be assumed for Socotran species until their boswellic acid content is measured and tested.

**Cytotoxic and antitumour evidence.** The most coherent preclinical dataset concerns *D. cinnabari* resin and oral squamous cell carcinoma, progressing from cell lines to two rat carcinogenesis protocols. Its limitations are a single research group, a single carcinogen model, crude extracts without identified active constituent, and no pharmacokinetic data linking oral doses to tissue exposure. Selectivity towards cancer cells is also uncertain: a resin extract was cytotoxic to non-malignant fibroblasts below 10 µg/mL [41], and another was more cytotoxic to non-cancerous HEK-293 cells than to A549 lung cancer cells [45]. For Socotran *Boswellia* and *Commiphora*, the cytotoxicity data concern mainly bark extracts screened against cancer cell lines without non-malignant comparators, and crude extracts of *B. socotrana* resin were only weakly cytotoxic to a non-malignant cell line. None of these data supports an anticancer claim in humans.

**Viral antigen assays, antiprotozoal and enzyme-inhibition assays.** Cell-culture studies have reported antiviral-related findings for bark extracts of *B. elongata*, *B. ameero*, *B. dioscoridis*, and *C. parvifolia*, although the viruses and experimental endpoints differed [38,39]. Among the Socotran resins, *D. cinnabari* resin extract and two isolated chalcones were evaluated in HepG2.2.15 cells, with reductions in HBV surface and e antigen production reported [34,38]. These antigen measurements do not directly establish inhibition of HBV DNA replication, and no in vivo antiviral efficacy has been demonstrated. The antiprotozoal activity of *D. cinnabari* resin was non-selective. The reported effects of the chalcones on HBV antigen production, acetylcholinesterase inhibition by *Boswellia* resin oils, and α-glucosidase inhibition by *B. elongata* triterpenoid acids remain based on individual in vitro studies [17,20,34].

## 8. Safety and Toxicological Evidence

Formal toxicity data were identified for only one of the three resin groups. For *D. cinnabari*, a guideline-based rat study reported no mortality or major adverse changes in the assessed endpoints following acute or 28-day oral administration of a resin methanol extract, although increased water intake was observed in treated animals [47]. These findings apply only to the tested extract and experimental conditions and do not establish the general safety of the whole resin, other preparations, longer-term exposure, or reproductive, developmental, genotoxic, topical, or ocular effects. Three additional safety-related issues warrant further investigation.

An ethnobotanical survey reported that 80% of informants believed *D. cinnabari* resin could induce abortion [2]. This represents a reported traditional belief, not an observed or experimentally verified effect. No study directly assessing reproductive or developmental toxicity of the resin was identified. Two isolated chalcones reported from *D. cinnabari*, 2-hydroxychalcone and, to a lesser extent, 4,6-dihydroxychalcone, inhibited CYP1A-dependent 7-ethoxyresorufin O-deethylase (EROD) activity in vitro using mouse liver microsomes [35]. No studies evaluating their effects on human CYP isoforms or assessing herb–drug interactions in vivo were identified; therefore, these findings indicate a potential interaction concern rather than an established clinical risk. An electrochemical prescreening study suggested a higher “potential carcinogenicity” index for one resin homoisoflavan [48], but this signal has not been followed by standard genotoxicity testing.

No toxicity study of a Socotran *Boswellia* or *Commiphora* resin and no human safety data for any of the three groups were identified in the literature searched. Safety information for commercial frankincense and myrrh obtained from other species, such as *C. myrrha*, is not applicable. Substitution of dragon’s blood sold in markets [2] raises a practical safety concern because the composition of purchased material may differ from that of authenticated resin.

## 9. Current Evidence Gaps and Future Research

**Botanical authentication and plant part.** Several frequently cited studies used bark rather than resin, and for some studies the plant part or material provenance could not be established. Future studies should report voucher specimens, collection site and date, and the organ sampled, and, for traded dragon’s blood, should confirm *D. cinnabari* origin, for example, by the 7,4′-dihydroxyflavylium chromophore [15]. Adopting *Boswellia* nomenclature based on the genus monograph and the subsequent formal descriptions of *B. hesperia* and *B. oropedionis* would reduce confusion between older and newer species concepts [23,24,32].

**Chemical standardization.** Yangambin has been quantified in *C. ornifolia* resin extracts at 3.50–9.05% (*w*/*w*) of the total ethanol extract [27], but validated multi-marker standardization is lacking for any Socotran resin. Priority targets are validated HPLC methods for the principal homoisoflavans and chalcones of *D. cinnabari*, boswellic and tirucallic acids in each Socotran *Boswellia* species, and yangambin and other non-volatile constituents in Socotran *Commiphora*.

**Variability and reproducibility.** Variation in yangambin content among eight *C. ornifolia* resin samples and differences in volatile profiles between two accessions of the species have been reported [26,27]. Together with the six-site investigation of *B. elongata* [21], these findings document chemical variability but do not establish reproducible, population-level chemotypes [21,26,27]. Future studies should therefore employ multi-site, multi-season sampling, standardised extraction and analytical protocols, retention indices, authentic standards, and quantitative marker analysis to distinguish genuine chemical variability from methodological artefacts. Biological assays should also report appropriate positive and solvent controls, replicate numbers, and relevant standardised methods.

**Mechanistic evidence.** Mechanistic data were identified only for the apoptotic effects of *D. cinnabari* resin extract in oral carcinoma cells [42,43]. Bioassay-guided isolation linked to target-level assays is needed to identify the constituents responsible for the reported activities of each resin.

**Toxicology.** Priorities are reproductive and developmental toxicity studies of *D. cinnabari* resin, given the traditional abortifacient report; standard genotoxicity testing of extracts and major constituents; dermal and ocular irritation studies matching traditional routes; and acute and repeated-dose studies of Socotran *Boswellia* and *Commiphora* resins.

**Traditional indications not yet tested.** Wound healing, haemostasis and gastrointestinal effects are the most frequently cited traditional uses but lack adequate experimental data for authenticated Socotran resin. They are logical candidates for well-designed in vivo studies with chemically characterized preparations.

**Human studies.** Clinical investigation would be premature until a defined resin preparation and an appropriate non-clinical evidence package are available. The preparation should be made from authenticated, voucher-documented material of a stated plant part and characterised by reproducible analytical profiles, with quantified marker compounds where appropriate and acceptable batch-to-batch consistency [50]. Its efficacy should be shown reproducibly in a preclinical model relevant to the intended indication, preferably with a dose–response assessment. The non-clinical safety package, including repeated-dose toxicity and genotoxicity, should match the intended route, duration and extent of exposure and the planned trial population [51]. Reproductive and developmental risks require particular attention if women of childbearing potential are to be enrolled [51], given the traditional belief that the resin can cause abortion [2]; this ethnobotanical report, however, does not establish reproductive toxicity. Exposure data should be sufficient to relate the proposed human dose to the preclinical findings. Topical use additionally requires assessment of dermal irritation, skin sensitisation, local tolerance and, where relevant, systemic absorption, whereas oral use requires a broader systemic safety and pharmacokinetic evaluation. Topical preparations of *D. cinnabari* resin, which correspond to its traditional use in wound care, may represent a reasonable starting point, provided that the specific preparation and indication are supported by adequate preclinical efficacy and safety data.

**Conservation and supply.** Resin-harvesting practices documented in the Firmihin forest were assessed as unsustainable [9], while overgrazing strongly impedes the natural regeneration of Socotran *Boswellia* [52]. These conservation concerns highlight the importance of sustainable sourcing. Any future development of these resins should prioritise sustainable harvesting, and research designs should minimise the amount of wild material collected.

## 10. Conclusions

The resins of *Dracaena cinnabari*, Socotran *Boswellia* and Socotran *Commiphora* have well-documented places in Socotran ethnomedicine, but the scientific evidence behind them is uneven and entirely preclinical. For *D. cinnabari*, the phenolic chemistry is well characterized, antifungal and radical-scavenging activities have been reported in vitro, a series of preclinical studies from one research group has examined oral carcinoma models, and a 28-day rat study provides limited oral toxicity data for a specific methanolic extract without establishing the general safety of the resin. For Socotran frankincense and Socotran *Commiphora* resins, available knowledge consists mainly of volatile profiles, two isolation studies and a few in vitro assays, while most of the biological data for these genera concern bark.

The evidence does not support any therapeutic claim in humans for any of the three resin groups, and it does not support transferring the pharmacology of *B. serrata*, *B. sacra*, *C. myrrha* or other sources of dragon’s blood to Socotran species. The most useful next steps are authenticated sampling, quantitative chemical profiling and targeted toxicology, followed by in vivo testing of the traditional wound-related and gastrointestinal uses with standardized preparations.

## Figures and Tables

**Table 2:** Experimentally reported biological activities of identified Socotran material. Studies are in vitro unless stated otherwise; no eligible human study was identified.

Taxon	Material	Activity	Model	Principal Finding	References
*D. cinnabari*	Methanolic solution of resin	Antifungal	Disc diffusion; MIC	Zones 20–30 mm against dermatophytes and *A. fumigatus*; MIC 500 µg/mL	[2]
*D. cinnabari*	Chloroform and methanol resin extracts; leaf extracts	Antibacterial	Agar diffusion, 4 mg/disc	Resin: zones 10–22 mm (incl. 6 mm disc) vs. Gram-positive bacteria incl. multiresistant staphylococci; chloroform extract 12 mm vs. *E. coli*; leaves inactive	[36]
*D. cinnabari*	Ethanolic resin extract	Antimicrobial	Disc diffusion	Zones 4.9–11.5 mm	[37]
*D. cinnabari*	Methanolic solution of resin	Radical scavenging; cytotoxicity	DPPH; FL cells	IC_50_ 25.5 µg/mL (ascorbic acid 15.5 µg/mL); FL cell IC_50_ 300 µg/mL	[2]
*D. cinnabari*	Isolated chalcones from resin	HBV antigen production	HepG2.2.15 cells	HBsAg IC_50_ 20.56 and 6.36 µg/mL	[34]
*D. cinnabari*	Resin methanol extract	HBV antigen production	HepG2.2.15 cells	HBsAg IC_50_ 20.93 µg/mL; CC_50_ 288.24 µg/mL	[38]
*D. cinnabari*	Resin methanol extract	Antiprotozoal; cytotoxicity	*P. falciparum*, *T. cruzi*, *T. brucei*, *L. infantum*; MRC-5 fibroblasts	IC_50_ 2.1–8.1 µg/mL; MRC-5 IC_50_ 7.7 µg/mL (non-selective)	[41]
*D. cinnabari*	Resin methanol extract	Cytotoxicity	A549 lung cancer and HEK-293 cells, 24 h	IC_50_ 90.96 (A549) vs. 72.10 µg/mL (HEK-293)	[45]
*D. cinnabari*	Resin methanol extract and fractions	Cytotoxic, apoptotic	Six OSCC cell lines; normal oral fibroblasts	Extract IC_50_ 5.9–19.2 µg/mL; fibroblasts not inhibited at 30 µg/mL; intrinsic apoptosis, S-phase arrest	[42]
*D. cinnabari*	Resin methanol extract	Antiproliferative, apoptotic	H103 cells; normal oral fibroblasts	IC_50_ 5.5 µg/mL (H103); fibroblasts not inhibited at 30 µg/mL; S- and G2/M-phase arrest; reduced migration; intrinsic apoptosis	[43]
*D. cinnabari*	Resin methanol extract, 100–1000 mg/kg orally	Chemopreventive, co-administered with carcinogen (animal)	4NQO oral carcinogenesis, rats	OSCC incidence 42.9%, 28.6% and 14.3% vs. 85.7% (*n* = 7 per group); reduced cyclin D1, Ki-67, Bcl-2 and p53 at 1000 mg/kg	[44]
*D. cinnabari*	Resin methanol extract, 100–1000 mg/kg orally for 10 weeks after carcinogen exposure	Post-initiation treatment (animal)	4NQO oral carcinogenesis, rats	SCC incidence 57.1%, 28.6% and 14.3% vs. 85.7% (*n* = 7 per group)	[43]
*B. elongata*	Bark methanol extract	Antibacterial; antiviral	Agar diffusion; influenza A and HSV-1 in cell culture	Active against Gram-positive bacteria including multiresistant *Staphylococcus*; IC_50_ 3.1 µg/mL (influenza A), 0.35 µg/mL (HSV-1)	[36,39]
*B. ameero*	Bark methanol extract	Antibacterial	Agar diffusion	Active against Gram-positive bacteria including multiresistant *Staphylococcus*	[36]
*B. ameero*	Bark methanol extract	Antiviral	Influenza A and HSV-1 in cell culture	IC_50_ 12.5 µg/mL (influenza A), 1.5 µg/mL (HSV-1)	[39]
*B. elongata*, *B. ameero*	Bark methanol extracts	Antiproliferative	Five human cancer cell lines	IC_50_ > 50 µg/mL (inactive)	[40]
*B. socotrana*, *B. dioscoridis*	Bark methanol extracts	Antimicrobial; radical scavenging; antiproliferative	Disc diffusion, MIC; DPPH; 5637, A-427, MCF-7 cells	MIC 125 µg/mL (*S. aureus*, *M. flavus*); 88–90% DPPH at 50 µg/mL; IC_50_ 18.4–29.0 µg/mL (5637, A-427), >50 µg/mL (MCF-7)	[49]
*B. socotrana*	Bark methanol extract	Antiprotozoal	*T. cruzi*, *T. brucei*; MRC-5 fibroblasts	IC_50_ 8.3 and 9.3 µg/mL; SI 3.9 and 3.5	[41]
*B. dioscoridis*, *B. socotrana*	Bark methanol extracts	HBV antigen production	HepG2.2.15 cells	IC_50_ 24.5 µg/mL (TI 14.8) and 118.9 µg/mL (TI 2.4)	[38]
*B. elongata*	Bark methanol extract, 200 and 400 mg/kg orally	Anti-inflammatory; antinociceptive (animal)	Carrageenan paw oedema and cotton pellet granuloma (rats); writhing and hot plate (mice)	At 400 mg/kg: oedema reduced by 82%, granuloma weight by 51%, writhing by 67%	[18]
*B. elongata*, *B. socotrana*, *B. dioscoridis*	Bark essential oils	Antimicrobial	Broth microdilution	Mainly Gram-positive; MIC 1.87–17.25 mg/mL; weak DPPH scavenging	[19]
*B. socotrana*	Aqueous, methanol and chloroform resin extracts	Cytotoxicity	FL cells (non-malignant), neutral red uptake	IC_50_ 454.6–535.1 µg/mL	[22]
*B. elongata*, *B. ameero*, *B. socotrana*	Resin essential oils	Radical scavenging; acetylcholinesterase inhibition	DPPH; Ellman assay	IC_50_ 121.4–211.2 µg/mL; 29.6–59.3% inhibition at 200 µg/mL	[17]
*B. elongata*	Isolated resin triterpenoid acids	α-Glucosidase inhibition	Yeast α-glucosidase assay; enzyme kinetics (experimental); molecular docking (computational support only)	IC_50_ 9.9–56.8 µM; non-competitive	[20]
*C. ornifolia*, *C. parvifolia*	Bark essential oils	Antimicrobial	Broth microdilution	Mainly Gram-positive; MIC 0.40–3.25 mg/mL (*C. ornifolia*) and 2.18–8.75 mg/mL (*C. parvifolia*)	[25]
*C. parvifolia*	Bark methanol extract	Antiviral	Influenza A and HSV-1 in cell culture	IC_50_ 13.0 µg/mL (HSV-1); inactive against influenza A	[39]
*C. parvifolia*	Bark methanol extract	Antibacterial	Agar diffusion	Active against Gram-positive bacteria including multiresistant *Staphylococcus*	[36]
*C. parvifolia*	Bark methanol extract	Antiproliferative	Five human cancer cell lines	IC_50_ 41.7 µg/mL (5637); >50 µg/mL (others)	[40]
*C. ornifolia*	Bark methanol extract	Antimicrobial; radical scavenging; antiproliferative; HBV antigen assay	Disc diffusion, MIC; DPPH; three cancer cell lines; HepG2.2.15	MIC 250 µg/mL; 86% DPPH at 50 µg/mL; IC_50_ 30.2–38.5 µg/mL; no HBsAg inhibition reported	[38,49]
*C. ornifolia*	Aqueous, methanol and chloroform bark extracts	Cytotoxicity	FL cells (non-malignant), neutral red uptake	Chloroform extract IC_50_ 39.3 µg/mL; aqueous and methanol extracts above 450 µg/mL	[22]

Abbreviations: 4NQO, 4-nitroquinoline-1-oxide; CC_50_, 50% cytotoxic concentration; DPPH, 2,2-diphenyl-1-picrylhydrazyl; FL, human amniotic epithelial cell line; HBsAg, hepatitis B surface antigen; HBV, hepatitis B virus; HSV-1, herpes simplex virus type 1; IC_50_, half-maximal inhibitory concentration; MIC, minimum inhibitory concentration; OSCC, oral squamous cell carcinoma; SCC, squamous cell carcinoma; SI, selectivity index; TI, therapeutic index; G2/M, gap 2/mitosis phase; *n*, number of animals per group. Bark-derived findings are included for contextual comparison only and should not be interpreted as evidence of biological activity of the corresponding resins.

**Table 3:** Selected constituents of Socotran resins: chemical characterization and biological-testing evidence.

Compound Identified (Class)	Botanical Source	Plant Part/Preparation	Amount/Concentration and Basis	Biological Assay	Reported Result/Activity	Evidence Level	Activity Demonstrated with Socotran Material?	Ref.
Homoisoflavans (e.g., 7,8-methylenedioxy-3-(4-hydroxybenzyl) chromane) ^a^	*D. cinnabari*	Resin-derived isolates	No concentration in whole resin reported	Mouse liver microsomes: Fe^2+^/NADPH lipid-peroxidation assay	A homoisoflavan inhibited lipid peroxidation, reported comparable to quercetin; H1/H2 identity inconsistently labelled in [35] ^a^	Compounds isolated; in vitro microsomal assay	Unverified—endemic Socotran taxon; collection provenance of the tested material not reported in [35].	[11,35]
Chalcones (2-hydroxychalcone; 4,6-dihydroxychalcone)	*D. cinnabari*	Resin-derived isolates	No concentration in whole resin reported	Mouse liver microsomes: CYP1A-dependent EROD and lipid-peroxidation assays	2-Hydroxychalcone inhibited EROD strongly; 4,6-dihydroxychalcone less strongly. Both were weak in lipid-peroxidation assay.	Isolated; in vitro microsomal assays	Unverified—endemic Socotran taxon; collection provenance of the tested material not reported in [35].	[35]
2,4′-Dihydroxy-4-methoxydihydrochalcone; 2,4′-dihydroxy-4-methoxyhydrochalcone	*D. cinnabari*	Resin/methanol extract	90 mg and 4 mg isolated from 3.0 g methanol extract, respectively (not whole-resin content)	HepG2.2.15 cell culture: HBsAg/HBeAg measurements	HBsAg IC_50_: 20.56/6.36 µg/mL; CC_50_: 346.67/242.11 µg/mL (in compound order). No HBV DNA measurement.	Isolated; in vitro cell-based surrogate endpoints	Yes; resin from Socotra reported in [34].	[34]
Lophenol and related sterols/triterpenoids	*D. cinnabari*	Resin/hexane extract fractions	Lophenol called dominant (86%); denominator not specified—NOT 86% of resin	No biological assay in cited study	Chemical identification only; no demonstrated bioactivity in [12].	GC and GC–MS as acetates in fractions; lophenol not individually purified	Not applicable (no activity test)	[12]
Cinnabarone (biflavonoid)	*D. cinnabari*	Resin/methanol extract	Isolation yield 0.66% reported; basis unspecified (not whole-resin concentration)	No biological assay in cited study	Biological activity not established in [10].	Isolated and structurally characterized	Not applicable (no activity test)	[10]
Damalachawin (triflavonoid)	*D. cinnabari*	Resin/methanol extract	Isolation yield 0.04% reported; basis unspecified (not whole-resin concentration)	No biological assay in cited study	Biological activity not established in [13].	Isolated and structurally characterized	Not applicable (no activity test)	[13]
Dracophane (metacyclophane)	*D. cinnabari*	Resin	Whole-resin concentration not reported	No biological assay in cited study	Biological activity not established in [14].	Isolated and structurally characterized	Not applicable (no activity test)	[14]
7,4′-Dihydroxyflavylium (chromophore)	*D. cinnabari*	Resin/colourant fraction	5–15% relative HPLC–DAD peak area of red colourants (12 resin samples); NOT whole-resin mass %	HPLC–DAD authentication; not a pharmacological assay	Proposed marker for *D. cinnabari*; biological activity not tested in [15].	Chromophore identified and profiled; relative-area measure	Not applicable (no activity test)	[15]
Boswellic and tirucallane acids (including KBA, AKBA, and the four triterpenoid acids named in Section 4.5)	*B. elongata*	Oleo-gum resin/methanol extract	Individual content in whole resin not quantified	Yeast α-glucosidase: primary screen at 1.0 mM; dose–response on four active compounds	IC_50_ (µM): 3α-Acetoxytirucall-8,24-dien-21-oic acid = 56.8; 3-O-acetyl-9,11-dehydro-β-boswellic acid = 9.9; 9,11-dehydro-β-boswellic acid = 20.9; 11α-methoxy-β-boswellic acid = 14.9. Values do not apply to KBA or AKBA. ^b^	21 isolates characterized; four with compound-specific in vitro IC_50_	Yes, for the four triterpenoid acids named in Section 4.5 (in vitro); no compound-specific IC_50_ reported for KBA or AKBA in [20].	[20]
Incensole (cembranoid diterpene)	*B. elongata*	Resin-derived isolate	Whole-resin content not quantified	Compound-specific α-glucosidase potency not reported	No compound-specific IC_50_ or established activity in [20]; screening description differs within source ^b^	Isolated; biological effect not resolved in cited study	Not established for the isolated compound	[20]
(+)-Yangambin (furofuran lignan)	*C. ornifolia*	Resin/recovered ethanol extract	3.50 ± 0.02 to 9.05 ± 0.19% (*w*/*w*) of recovered ethanol extracts; mean ± SD (*n* = 3); NOT original dry-resin %	No biological assay of isolated Socotran yangambin in cited study	Activity not established in [27].	Isolated; HPLC–DAD quantified in 8 resin extracts	Not applicable (no activity test)	[27]

Notes: HBsAg, hepatitis B surface antigen; HBeAg, hepatitis B e antigen; HBV, hepatitis B virus; IC_50_, half-maximal inhibitory concentration; CC_50_, 50% cytotoxic concentration; CYP1A, cytochrome P450 1A; EROD, 7-ethoxyresorufin O-deethylase; GC–MS, gas chromatography–mass spectrometry; HPLC–DAD, high-performance liquid chromatography with diode-array detection; KBA, 11-keto-β-boswellic acid; AKBA, 3-O-acetyl-11-keto-β-boswellic acid; SD, standard deviation. Reported percentages do not necessarily represent concentrations in the whole resin; the analytical basis is specified where available. Absence of reported biological testing does not establish inactivity. ^a^ Machala et al. [35] identify the active homoisoflavan as H2 in the Abstract but H1 in the Discussion; compound-specific attribution is therefore uncertain. ^b^ Ur Rehman et al. [20] state that all 21 isolates were screened at 1.0 mM, whereas the Abstract and Table 1 report eight. Compound-specific IC_50_ values are provided only for the four triterpenoid acids named in Section 4.5; their absence for other compounds does not establish inactivity.

## Data Availability

No new data were created or analyzed in this study. Data sharing is not applicable to this article.
